# Optimizing the Patient Telemedicine Experience in an Orthopaedic Clinic

**DOI:** 10.7759/cureus.16879

**Published:** 2021-08-04

**Authors:** Margaret A Sinkler, Joshua D Dolan, Michael J Steflik, Peter Harimtepathip, MD, J. Shell Cox, Stephen A Parada, MD

**Affiliations:** 1 Orthopaedics, Augusta University Medical College of Georgia, Augusta, USA; 2 Orthopaedic Surgery, Augusta University Medical College of Georgia, Augusta, USA

**Keywords:** telemedicine in orthopedics, telemedicine experience, orthopedic telemedicine, covid-19 telemedicine, covid-19 orthopedics, orthopedic visits, orthopedics, optimization

## Abstract

With the rapid advancements in today’s technology, the telemedicine model of healthcare has become an increasingly useful tool for healthcare providers and patients to interact outside of the confines of a traditional office visit. As a result of the COVID-19 pandemic, many providers have been forced to adopt a component of telemedicine into their practice. In an effort to improve the telemedicine system for continued use, 519 patients in an orthopaedic clinic at a Level One academic system were surveyed on their willingness and confidence to use telemedicine in future orthopaedic visits. Though most patients reported that they had been unwilling to use telemedicine for their current visit, the majority were neutral or willing to use telemedicine in the future. In this study, we present some challenges to the orthopaedic telemedicine visit, patient sentiment towards the current and future use of telemedicine in orthopaedics, as well as possible direction for improvement so that telemedicine can be better incorporated into the orthopaedic clinic.

## Introduction

The patient experience has been defined as “everything we say and do that affects our patients' thoughts, feelings, and well-being”, and is increasingly being recognized as a fundamental component of value-based care. Clinical studies have demonstrated that improvements to the patient experience yield greater satisfaction, increased adherence to treatment, and a maintained level of clinical outcomes [1-4]. 

With the advent of the novel coronavirus disease (COVID-19), virtual and off-site patient encounters have drastically increased [5]. In order to curb the spread of the virus, the Center for Disease Control and Prevention (CDC) recommended social distancing guidelines and limitation of physical contact between individuals in close proximity [6]. Telemedicine was first discussed and implemented as a concrete solution to continue medical care in a socially distanced setting during the West Africa Ebola outbreak in 2015 [7]. In a survey conducted of all the enhanced recovery after surgery (ERAS) orthopaedic programs, it was seen that 106 of the 168 respondents utilize telehealth services. Of these, 83% state that the COVID-19 pandemic was the impetus for the implementation of the service [8]. 

The telemedicine model protects both patients and clinicians from potential exposure, is conducive to self-quarantine, and is a 21st-century approach to triage patients [9]. The model has been successfully implemented largely through technological improvements, high-speed internet, and access to smartphones [7]. Many patients have had, or will have, their first telemedicine visit with a provider because of COVID-19. Although telemedicine servers have been largely implemented in the wake of the pandemic, there is need for discussing patient preferences and optimizing the system for continued use moving forward.

The purpose of this study is to evaluate patients’ current perception and to identify current limitations of telemedicine visits in an orthopaedic clinic. The specific aim of the study is to identify patient preferences related to telemedicine, willingness to participate, and the device that they intend to use. By gathering this information, we can optimize the telemedicine experience moving forward to ensure the best possible patient experience in the future.

## Materials and methods

Following Institutional Review Board (IRB) approval, informed consent and patient survey forms were distributed by front desk members at an outpatient orthopaedic surgery clinic upon arrival for in-person consultation from 11/23/2020 to 1/19/2021. If patients agreed to the consent, surveys were completed in full alongside intake forms prior to their orthopaedic consult. The questionnaires were collected by an authorized investigator and stored on a secured cloud drive. The inclusion criteria for the study were males and females presenting to the orthopaedic clinic at the institution for either first visit or recheck, who were above the age of 18 years and were capable of consenting to the study. Participation in the study was completely voluntary and had no impact on services rendered during the physical visit in which they were given the survey. Of the 640 surveys that were handed out, 516 were completed and eligible for inclusion in the survey. No direct or indirect patient identifiers were collected, and all 18 characteristics of the Health Insurance Portability and Accountability Act (HIPAA) de-identification standard were met. 

The Appendix includes the 11-question survey that was given to patients in the clinic. Responses were analyzed using a Mann-Whitney U Test in cases of non-parametric data or a Chi-square analysis in the case of categorical data. The R 4.0.0™ statistical package was used for statistical calculation. 

## Results

A total of 519 surveys were obtained from outpatient orthopaedic clinics between 11/23/2020 to 1/19/2021 with an 80.6% survey completion rate. A summary of respondent demographics may be found in Table 1. The full survey questionnaire is provided in the Appendix.

**Table 1:** Demographic information of survey participants

Question	Answer	n (%)
Sex	Male	203 (40.03)
	Female	304 (59.96)
Education	Less than high school	22 (4.26)
	High school	113 (21.90)
	Some college	147 (28.49)
	College	110 (21.32)
	Graduate degree or above	112 (21.71)
Median Age	54	

**Figure 1:**
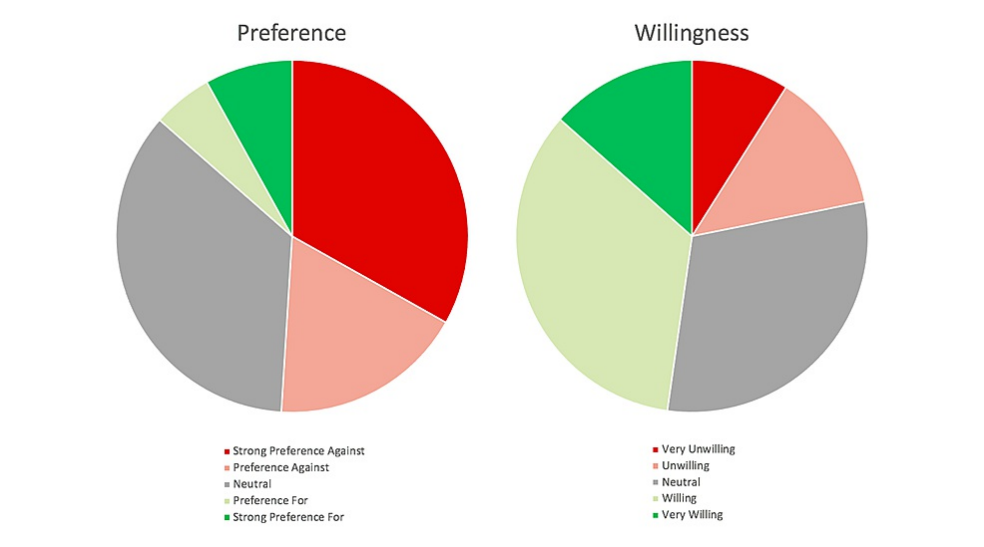
Percentage breakdown of surveyed patients demonstrating their preference and willingness to participate in telemedicine visits. After recording patient responses to the survey given prior to their appointment, their preference for a telemedicine visit was compared to their willingness to participate in a future telemedicine appointment. The chart on the left illustrates current patient sentiment regarding their preference for a telemedicine appointment versus an in-person appointment at the time of the survey. The results point to a majority preferring an in-person visit versus telemedicine. The chart on the right shows patient willingness to use telemedicine in the future and shows a dramatic increase in the number of patients willing to use telemedicine moving forward.

Over three-fourth (86.5%) of the orthopaedic patients surveyed were neutral or opposed to current telemedicine visits while only 13.5% (69) surveyed patients had a preference or strong preference for telemedicine visits. Approximately 33.1% of patients had a strong in-person preference and 8.0% had a strong preference for telemedicine (Figure 1). However, 78.2% (401) of patients possessed a neutral or willing outlook on future telemedicine visits compared to 21.8% (112) that were either unwilling or very unwilling to participate in future telemedicine visits (Figure 1). 

**Figure 2:**
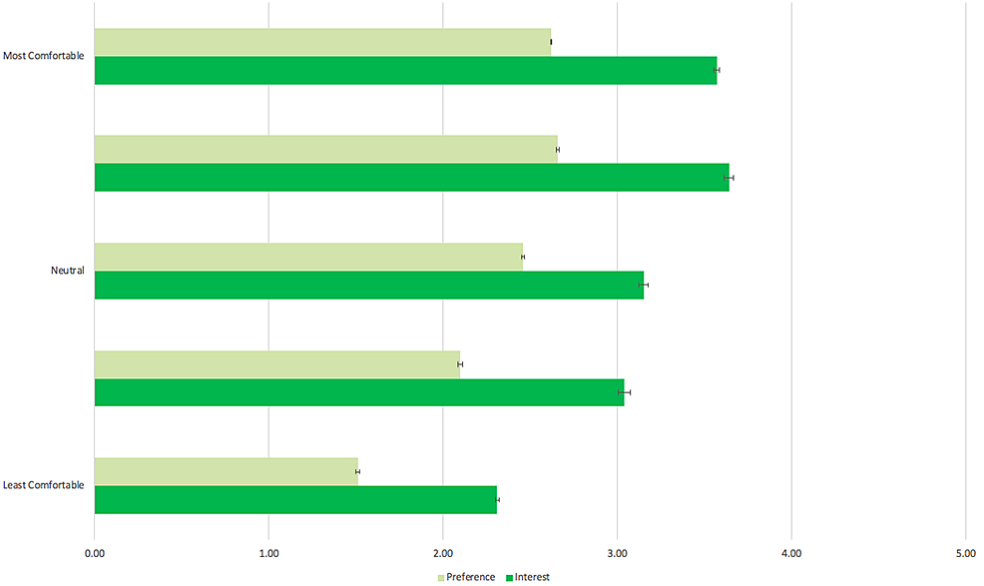
Relationship between patient preference or interest in future telemedicine visits versus their comfort with electronic devices. The horizontal axis denotes levels of patient preference for telemedicine visits and interest in future telemedicine visits in light green and dark green, respectively. 1 = very unwilling/strong preference for in-person visit; 2 = unwilling/slight preference for in-person visits; 3 = neutral; 4 = willing/slight preference for telemedicine; 5 = very willing/strong preference for telemedicine. The vertical axis represents levels of patient comfort with using their electronic devices for telemedicine. Both preference and future interest for telemedicine increase as patient comfort level with electronic devices increases. Error bars in all data points are based off of 95% confidence intervals.

Patient comfort level using electronic devices was directly related to their willingness for future telemedicine visits as well as the likelihood they preferred virtual visits currently (p < 0.05) shown in Figure 2. Exceptions to this include those who reported slight discomfort versus neutral comfort in using their electronic devices (p=0.50) and those who reported slightly comfortable versus those who reported extreme comfort using their electronic devices (p=0.68). Additionally, those less comfortable with their electronic devices expressed a strong preference for in-person visits while those who reported being more comfortable with their devices expressed a neutral preference for in-person versus telemedicine visits. This trend is significant except for when comparing those who reported neutral comfort with their devices with those who reported slight discomfort, slight comfort, and extreme comfort with their devices (p=0.09, p=0.48, p=0.70, respectively). There was also no statistical difference in current preference between those who expressed slight and extreme comfort (p=0.67). 

The majority of patients have had no direct or familial experience with prior telemedicine visits (74%). Those with prior experiences with telemedicine expressed greater willingness to participate in future telemedicine visits versus those with no history (p=3.97x10^-5). Those with a history of telemedicine visits had more neutral preferences for in-person versus telemedicine visits as opposed to those with no history of telemedicine, who had a stronger in-person visit preference (p=8.00x10^-5). 

The majority of patients reported that they would use a cell phone as their primary device (63.75%) followed by computer (15.41%), laptop (13.29%), and tablet (12.89%). A majority of respondents reported a second available device (65.85%) (Table 2). Those without stable or reliable internet were found to have a more neutral opinion on current visit type compared to those with Wi-Fi, who expressed greater preference for telemedicine (p<0.05). There was also a significant difference when comparing those without reliable Wi-Fi, who were less interested in the future of telemedicine visits, compared to those with access to reliable Wi-Fi, who were more neutral (p<0.05). 

**Table 2:** Device used for telemedicine visits by survey respondents

Primary Device	n (%)
Tablet	25 (7.55)
Laptop	44 (13.29)
Computer	51 (15.41)
Cell phone	211 (63.75)
Secondary Device	n (%)
Tablet	62 (21.83)
Laptop	53 (18.66)
Computer	62 (21.83)
Cell phone	81 (28.52)
Not specified	26 (9.15)
No secondary device	97 (34.15)

## Discussion

The novel coronavirus disease (COVID-19) pandemic had an immediate impact on in-person evaluation of patients, which has been demonstrated in multiple studies across the United States (US). A study conducted in an outpatient orthopaedic spine clinic in Utah during the initial four weeks of the COVID-19 pandemic, starting from March 16, 2020, showed that overall clinic visits decreased from 417 to 322 and new patient visits decreased from 28% to 20%. Of the 322 visits, 318 (98.7%) were performed via telemedicine [10]. In a survey given to the 3,400 members of the American Association of Hip and Knee Surgeons (AAHKS), starting March 20, 2020, over 90% of the surgeons surveyed reported a reduction in patient load in the first three months. Over time, the reported reduction in clinic volume dropped to 73% by mid-June, 2020, and 59% by September 2020. By September 2020, 35% of respondents were using telemedicine [11]. Therefore, the adoption and incorporation of telemedicine into practice had a large impact on maintaining clinic volumes during the COVID-19 pandemic. 

Telemedicine is not a new concept in medicine as initial manuscripts appeared in the late 19th century. Even prior to COVID-19, improvements in technology and devices had increased the relevance of telemedicine visits. Interest in telemedicine was evident earlier too, as the Kaiser Family Foundation found increasing rates of employee telemedicine coverage in 2019 [12]. While there are known benefits to telemedicine visits including increased access, lower cost, and high patient satisfaction rates, several barriers exist preventing the widespread use of telemedicine visits including implementation cost, lack of awareness, inefficiencies, medicolegal liability implications, and difficulty in examining patients virtually [13]. 

Our study was performed at a Level One academic center in the Southeast. In a recent study using 175 ERAS participating orthopaedic programs, there was a nationwide increase in the use of telemedicine with 74.4% of programs in the Northeast, 69.6% of programs in the West, 66.1% in the South, and 45.7% in the Midwest [8]. The largest changes in telemedicine visits were correlated to COVID-19 hot spots. New York state saw a 550% increase, California a 900% increase, and Texas a 1200% increase [8]. Therefore, our study most accurately reflects the current patient perception of telemedicine visits in the Southeast region.

Within our study, a majority of patients reported feeling neutral to opposed with regard to telemedicine visits. The J.D. Power survey in 2019 showed that 29% of patients did not believe that telemedicine services were available to them and 37% did not know if their provider offered such services [14]. Patients who had a telemedicine visit in the past were more likely to be willing to participate in future telemedicine visits. This finding agrees with prior studies that have compared telemedicine studies to in-person visits in orthopaedic clinics in multiple regions of the US [15-19]. 

The majority of participants in our study identified that they would utilize a cell phone as their main device and their comfort with the device directly correlated to their willingness to partake in telemedicine visits. This can be used to optimize the virtual visit towards our patients based on the selected software. Additionally, high-quality video consultations require 4-10 Mbps of Wi-Fi [15]. With the rapid advancements in technology and internet availability today, this requirement becomes less and less of an issue.

Prior to COVID-19, a 2013 study at UC Irvine evaluated telemedicine use after total joint arthroplasty where 34 patients had a telemedicine follow-up and 44 patients had an in-person follow-up. Post-operative telemedicine visits yielded higher satisfaction rates [16]. Additionally, in 2015, a prospective randomized control trial at Vanderbilt using 24 patients in an orthopaedic trauma center showed no significant difference in patient satisfaction between telemedicine and in-person visits [17]. In 2019, a study of 167 patients at a pediatric fracture clinic in Pennsylvania found similar satisfaction levels between groups that received telemedicine or in-person care [18]. During the COVID-19 pandemic, a prospective randomized control study in Philadelphia in 2020 had 66 patients utilizing telemedicine for post-operative visits after rotator cuff repair. Patients demonstrated similar pain scores and overall higher satisfaction scores using telemedicine when compared to in-person visits [19]. These studies show that long term implementation of telemedicine visits in orthopaedic clinics is a reasonable and feasible option.

Future studies should shift towards implementing and validating a universal telemedicine model in orthopaedic clinics and evaluating the longevity of telemedicine once the COVID-19 pandemic resolves. Provider feedback and perceived telemedicine limitations will be another critical direction that should be incorporated towards the improvement of the platform. In 2020, the Cleveland Clinic Foundation proposed a universally applicable model using a centralized appointment desk and a virtual short questionnaire that assesses severity. Patients were then stratified into groups according to their problem with high-risk patients directed either to the ED or scheduled for a routine visit [20]. Using this telemedicine triage model, the ease and accessibility of telemedicine can be applied and paired with the necessary in-person appointments, as requested and optimized through physician feedback, to improve the quality of orthopaedic healthcare and improve time efficiency for both patient and provider.

Limitations to the study include a single point of survey, that is the surveys were gathered at one Level One academic training site, and therefore the results may vary when compared to other centers. The surveys were gathered during one time period, that is during the pandemic, and may not reflect opinions in telemedicine following the pandemic. The respondents were not required to complete all survey questions. The study did not include specific aspects of medical care received by the participants to better classify who would benefit from telemedicine visits. 

## Conclusions

In this study, we surveyed the patients of an orthopaedic clinic at a Level One academic training site to identify their preferences for current and future orthopaedic telemedicine appointments. Following the initial impact of COVID-19, many providers have incorporated an aspect of telemedicine into their practice, and it continues to remain a popular fixture even as the country opens up. Though more than half of the patients surveyed said they preferred an in-person appointment at the time of their in-person visit, almost 80% reported they were also either neutral or willing to use telemedicine as a supplemental form of orthopaedic appointment in the future. Our data show that patient confidence in handling their electronic device is a significant factor in both their preference and willingness to use telemedicine. The results of this study reflect a growing trend in the use of technology in medicine as a way to increase the efficiency and efficacy of care. Even though this study involved only one site in the Southeast and a narrow period of time and sentiment, similar results have been found in other major populations at different times. Moving forward, studies that incorporate these existing data should consider investigating the longevity of a streamlined, universal telemedicine platform for use in orthopaedics.

## Figures and Tables

**Figure 3:**
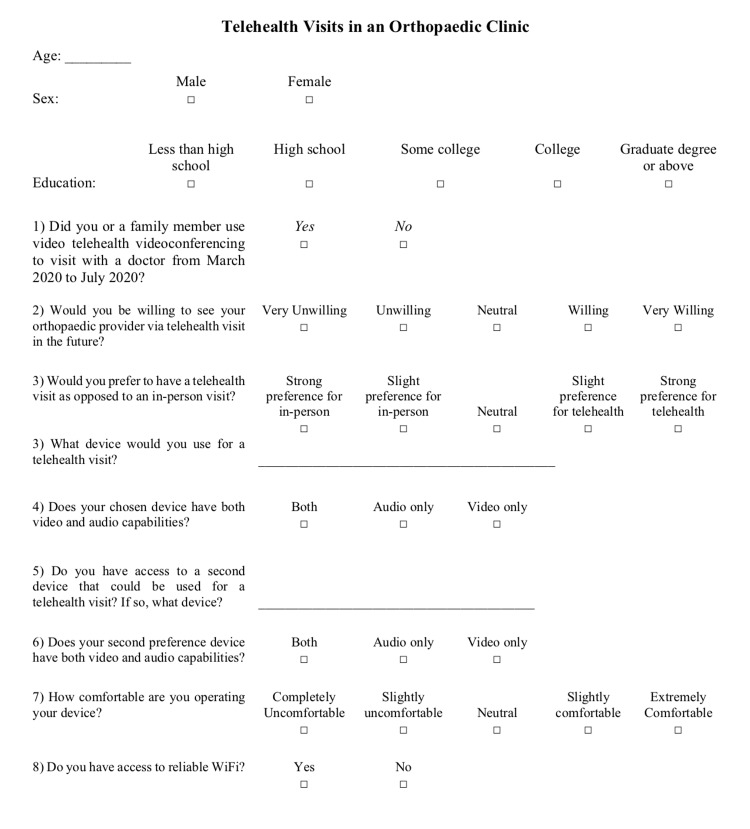
Telemedicine survey given to patients at the time of their visit.
